# Decoding YouTube: An In-depth Analysis of Living Donor Kidney Transplantation Videos and Their Implications for Patient Education

**DOI:** 10.1016/j.euros.2024.10.005

**Published:** 2024-10-19

**Authors:** Hakan Bahadir Haberal, Alberto Piana, Alessio Pecoraro, Beatriz Bañuelos Marco, Thomas Prudhomme, Alicia López-Abad, Marta Casadevall Rubau, Muhammet Irfan Donmez, Alberto Breda, Angelo Territo, the European Association of Urology Young Academic Urologists Kidney Transplantation Working Group

**Affiliations:** a Department of Urology, Ankara Ataturk Sanatoryum Training and Research Hospital, University of Health Sciences, Ankara, Turkey; b Department of Urology, San Luigi Gonzaga Hospital, University of Turin, Orbassano, Turin, Italy; c Unit of Urological Robotic Surgery and Renal Transplantation, Careggi Hospital, University of Florence, Florence, Italy; d Department of Urology, Renal Transplant Division, University Hospital Clínico San Carlos, Madrid, Spain; e Department of Urology and Kidney Transplantation, Rangueil University Hospital, Toulouse, France; f Department of Urology, Virgen de la Arrixaca University Hospital, Murcia, Spain; g Uro-oncology and Kidney Transplant Unit, Department of Urology, Fundació Puigvert Hospital, Autonoma University of Barcelona, Barcelona, Spain; h Istanbul Faculty of Medicine, Department of Urology, Division of Pediatric Urology, Istanbul University, Istanbul, Turkey

**Keywords:** Patient education, Instructional video, Kidney transplantation, Transplant recipients

## Abstract

**Background and objective:**

YouTube is an open online video platform that both patients and health care professionals use to access information. Our aim was to evaluate the quality of videos related to living-donor kidney transplantation (LDKT) on YouTube.

**Methods:**

Research was conducted using the keywords “living donor kidney transplant” and “kidney transplant”. We evaluated videos with more than 10 000 views and excluded those not in English. A total of 58 videos met the criteria for inclusion in the evaluation. We used the modified DISCERN tool, the Journal of the American Medical Association (JAMA) benchmark score, and the Global Quality Score (GQS) to evaluate the quality, accuracy, and educational value.

**Key findings and limitations:**

The quality of the videos was low, with a median DISCERN score of 1 (interquartile range [IQR] 1–2), JAMA score of 1 (IQR 1–2), and GQS of 2 (IQR 1–3). The majority of the videos were of North American origin (75.9%) and focused on the patient experience (51.7%). The scores for patient experience videos were significantly lower than for other videos according to all three scoring systems (*p* < 0.001). Videos uploaded by medical centers and private physicians/nurses had significantly higher scores than videos uploaded by patients or by TV shows/programs. Evaluation of only videos with English audio is a limitation of the study.

**Conclusions and clinical implications:**

Assessment of YouTube videos presenting information on LDKT revealed low quality. Health care organizations should create online resources and share them on social media platforms.

**Patient summary:**

We looked at the quality of YouTube videos on kidney transplantation using a kidney from a living donor. We found that the information presented in YouTube videos on this topic is of low quality.

## Take Home Message

We found that YouTube videos on living-donor kidney transplantation often contain substandard content and misinformation. Health care providers should create and share accurate educational materials on popular social media platforms for better patient access.

## 1 Introduction

It is well established that kidney transplantation (KT) is the most effective treatment method for end-stage renal failure, offering patients better ability to participate in activities of daily living in comparison to dialysis [1]. In regions where the availability of deceased donor organs is limited, living-donor KT (LDKT) has become a widespread practice, filling the critical gap in organ supply. However, lack of knowledge about LDKT along with the psychological complexity for donors may mean that appropriate treatment is not available for potential patients [2].

In the contemporary digital age, the proliferation of technology has led to unprecedented availability of information on online platforms. While this abundance of information is a boon, ensuring its quality and accuracy is paramount. Patients and health care professionals increasingly turn to online resources to gather insights into medical conditions and the treatment options available, with YouTube emerging as a prominent platform in this regard [3]. Given this context, it is crucial to assess the utility and reliability of YouTube videos on LDKT for patient information and education. As the demand for accurate medical knowledge surges, evaluation of the content available on this widely used platform is of significant importance. Such evaluations not only empower patients by ensuring that they receive reliable information but also aid health care professionals in guiding their patients towards trustworthy resources, enhancing the overall quality of patient care and education in the KT setting.

Previous studies have evaluated the content of online information platforms regarding different organ transplantations, donor surgeries, and other surgical procedures [4], [5], [6], [7], [8]. However, to the best of our knowledge, there has been no such evaluation of LDKT online content. Therefore, we conducted an evaluation of the content of videos on LDKT on the YouTube platform using validated questionnaires and scoring systems. Our aim was to assess the suitability of these videos for practical use in patient education. The study findings not only contribute to the assessment of online medical information but may also inform the development of educational strategies that empower patients to make well-informed decisions regarding LDKT.

## 2 Materials and methods

Research was conducted on YouTube using the keywords “living donor kidney transplant” and “kidney transplant” on December 17, 2023. First, we considered videos with a duration of 4–20 min, as we considered that this timeframe is optimal for engaging viewers while delivering substantial information. Videos with fewer than 10 000 views were not taken into account. This first search identified 211 videos for evaluation. We then excluded videos that were not in English to ensure that the content analyzed is accessible to a wide audience. If a video featured twice, only one of the videos was evaluated. The content of the videos spanned various aspects of KT, including explanations of the surgical procedure, postoperative follow-up care, and the advantages of undergoing KT. We included these specific themes to provide a comprehensive overview of LDKT, addressing both procedural process and the benefits to patients. The search results were compiled in a playlist and then two researchers (H.B.H. and A.P.) reviewed and analyzed the videos independently. Both surgeons separately scored the videos, and any discrepancies were addressed via discussion and clarification. Of the 211 videos initially identified, 153 were excluded for the following reasons: the content was not relevant (*n* = 38), the video was in a language other than English (*n* = 82), the video was related only to kidney donation (*n* = 21), the video had no audio (*n* = 6), the video was related to transplantation of another organ (*n* = 3), duplicate videos (*n* = 2), or the video had unlisted status on YouTube (*n* = 1).

For each video included in our study, a comprehensive set of data points were collected. The videos were classified into groups according to their content (pretransplantation preparation, transplantation surgical procedure, post-transplantation follow-up, personal experience, and general information related to KT), target audience (patients, physicians/nurses or general population), video source (medical center, physician/nurse, patient or television show/program), region of origin (Europe, North America, Asia, or Africa), and year uploaded (2008–2015 or 2016–2023). For each video, the numbers of views and likes, the upload date, video length (in seconds), duration on YouTube (in days), and view ratio (number of views/duration on YouTube) were collected.

We used a set of well-established criteria to assess the quality, accuracy, and educational value of the videos. These included the modified DISCERN tool, designed to evaluate the reliability of health information in online videos [9]. The original DISCERN tool comprises 16 questions, each rated on a scale from 1 to 5. By contrast, the modified DISCERN tool consists of five questions, with 1 point assigned for each aspect addressed in the video [9], [10]. We also used the Journal of the American Medical Association (JAMA) benchmark score, a recognized metric for assessing the authenticity, efficiency, and trustworthiness of medical information [11]. We used the Global Quality Score (GQS) to gauge the overall quality and effectiveness of the video content to ensure that viewers receive reliable and valuable information [12].

### 2.1 Statistical analysis

All statistical analyses were performed using SPSS 25.0 (IBM Corp., Armonk, NY, USA) for Windows. Statistical significance was set at *p* < 0.05. The distribution of variables was assessed using the Kolmogorov-Smirnov test. Results for categorical data are expressed as the frequency and percentage, while results for nonparametric variables are presented as the median and interquartile range (IQR). Results were compared using a Mann-Whitney U test or Kruskal-Wallis test. Spearman correlation analysis was used to evaluate the correlation between variables.

## 3 Results

A total of 58 videos met the criteria for inclusion in the evaluation. The majority of the videos were uploaded by medical centers (37.9%), followed by compared to physicians/nurses (15.5%), patients (25.9%), and television shows/programs (20.7%). In terms of country of origin, 75.9% of the videos were uploaded from North America. Analysis of video content revealed that patient experience videos (51.7%) were the most common, while videos on to the pretransplantation period (3.4%) were the rarest. The inter-rater reliability among researchers was κ = 0.554 (95% confidence interval [CI] 0.396–0.712) for DISCERN scores, κ = 0.725 (95% CI 0.543–0.907) for JAMA scores, and κ = 0.668 (95% CI 0.523–0.813) for the GQS scale. The median DISCERN, JAMA, and GQS scores were 1 (IQR 1–2), 1 (IQR 1–2), and 2 (IQR 1–3), respectively. Table 1 lists descriptive information and scores for the videos.

**Table 1:** Characteristics of the videos reviewed for this study

Parameter	Result
Video content, *n* (%)	
Pretransplantation	2 (3.4)
Surgery	6 (10.3)
Post-transplantation	7 (12.1)
Patient experience	30 (51.7)
General information	13 (22.4)
Uploader, *n* (%)	
Medical center	22 (37.9)
Private physician/nurse	9 (15.5)
Patient	15 (25.9)
TV show/programme	12 (20.7)
Target audience, *n* (%)	
Physician/nurse	8 (13.8)
Patient	22 (37.9)
General population	28 (48.3)
Country of origin, *n* (%)	
North America	44 (75.9)
Europe	6 (10.3)
Asia	6 (10.3)
Africa	2 (3.4)
Year uploaded, *n* (%)	
2008–2015	16 (27.6)
2016–2023	42 (72.4)
Median video length, s (IQR)	449 (314.5–655.75)
Median time since uploaded, d (IQR)	2003 (1264.75–3086.75)
Median number of views (IQR)	44 432 (19 162.75–140 975.75)
Median number of likes (IQR)	501 (231.25–1025)
Median view ratio (IQR)	27 (11.7–84.35)
Median DISCERN score (IQR)	1 (1–2)
Median JAMA score (IQR)	1 (1–2)
Median Global Quality Score (IQR)	2 (1–3)

IQR = interquartile range; JAMA = Journal of the American Medical Association.

There were significant differences in scores for LDKT videos across the content categories of pretransplantation preparation, transplantation surgical procedure, post-transplantation follow-up, personal experience, and general information (DISCERN, *p* < 0.001; JAMA, *p* = 0.001; GQS, *p* < 0.001). Subgroup analysis revealed that patient experience videos had significantly lower scores than videos in the other categories. Therefore, we compared the patient experience category to a pooled group comprising all the other video categories. The results for this comparison revealed that the scores for patient experience videos were significantly lower than for the pooled group of other categories using all three scoring systems (*p* < 0.001).

There were statistically significant differences in scores across the uploader categories (*p* < 0.001 for all scoring systems). Subgroup analysis revealed that videos uploaded by medical centers and physicians/nurses had significantly higher scores than patient and TV show/program videos.

There were significant differences in scores across the target audience categories (*p* < 0.001 for all scoring systems). Subgroup analysis revealed higher scores for videos tailored for physicians/nurses and patients than for videos targeted at the general population.

There were significant differences in JAMA (*p* = 0.015) and GQS (*p* = 0.046) scores for video origin. Further analysis videos originating from North America had significantly higher GQS scores than those from Europe (*p* = 0.033).

There were no significant differences in the scores for year of uploading (DISCERN, *p* = 0.622; JAMA, *p* = 0.843; GQS, *p* = 0.158). The results are listed in Table 2.

**Table 2:** Comparison of scores for video characteristics

Parameter	Median DISCERN score (IQR)	*p* value	Median JAMA score (IQR)	*p* value	Median GQS (IQR)	*p* value
Video content		**<0.001**^a^		**0.001**^a^		**<0.001**^a^
Pretransplantation^c^	3		1.5		3	
Surgery	2 (0)		2 (0)		3 (2)	
Post-transplantation	2 (1)		1 (1)		2 (0)	
Patient experience	1 (1)		1 (0)		1.5 (1)	
General information	2 (1)		2 (1)		3 (2)	
Uploader		**<0.001**^a^		**<0.001**^a^		**<0.001**^a^
Medical center	2 (1)		2 (1)		3 (2)	
Physician/nurse	2 (0)		1 (1)		2 (1)	
Patient	1 (1)		1 (0)		2 (1)	
TV show/program	1 (2)		1 (0)		1 (1)	
Target audience		**<0.001**^a^		**0.001**^a^		**<0.001**^a^
Physician/nurse	2 (1)		2 (1)		3 (2)	
Patient	2 (1)		1 (1)		2 (1)	
General population	1 (1)		1 (0)		1.5 (1)	
Country of origin		0.053^a^		**0.015**^a^		**0.046**^a^
North America	2 (1)		2 (1)		3 (2)	
Europe	1 (1)		1 (0)		2 (2)	
Asia	2 (1)		2 (1)		2.5 (1)	
Africa^c^	1		1		1.5	
Year uploaded		0.622^b^		0.843^b^		0.158^b^
2008–2015	1.5 (2)		1 (1)		2 (1)	
2016–2023	1 (1)		1 (1)		2 (2)	

GQS = Global Quality Score; IQR = interquartile range; JAMA = Journal of the American Medical Association.

a Kruskal-Wallis test.

b Mann-Whitney U test.

c No IQR is provided as there were only two data points.

Significant positive correlations were observed between DISCERN, JAMA, and GQS scores. However, there were no significant correlations between video length, view ratio, like count, and the three scores (Table 3).

**Table 3:** Spearman analysis of correlation between scoring systems, video length, view ratio, and like count

	DISCERN score	JAMA score	Global Quality Score
DISCERN score	–	***r*** = **0.551** ***p*** < **0.001**	***r*** = **0.769** ***p*** < **0.001**
JAMA score	***r*** = **0.551** ***p*** < **0.001**	–	***r*** = **0.620** ***p*** < **0.001**
Global Quality Score	***r*** = **0.769** ***p*** < **0.001**	***r*** = **0.620** ***p*** < **0.001**	–
Video length	*r* = 0.050 *p* = 0.710	*r* = −0.175 *p* = 0.188	*r* = −0.034 *p* = 0.798
View ratio	*r* = 0.099 *p* = 0.462	*r* = −0.053 *p* = 0.692	*r* = −0.098 *p* = 0.462
Like count	*r* = 0.024 *p* = 0.860	*r* = −0.092 *p* = 0.494	*r* = −0.128 *p* = 0.339

JAMA = Journal of the American Medical Association.

## 4 Discussion

Social media platforms now play a pivotal role as vital conduits for health care professionals and patients alike, facilitating seamless access to information and fostering interactive exchanges [13]. Following the establishment of YouTube, studies evaluating videos related to health issues began in 2007 and have gradually increased overall and in the field of urology [7], [8], [14], [15], [16], [17]. While the accuracy and reliability of YouTube videos on various organ transplant and donor surgeries have been evaluated in previous studies, there is a notable lack of studies on LDKT videos [4], [5], [6]. This research gap is particularly conspicuous considering the pivotal role of LDKT, especially in regions where there is a scarcity of cadaveric organs. Thus, our aim was to assess the quality of videos on LDKT, focusing our attention specifically on YouTube. Our results show that the quality and educational level of LDKT videos available on YouTube are low.

Our analysis according to specified keywords on YouTube led to exclusion of 153 videos from the evaluation process. Videos were excluded because of lack of relevance to the topic or the use of languages other than English, which highlights the challenges faced by this patient population in accessing information about their disease and treatment options via social media channels. The predominant languages in the majority of videos excluded because they were not in English were Hindi and Urdu. While this undeniably provides an advantage for users proficient in Hindi or Urdu, regrettably, these videos are not accessible to the global audience. Some of the videos included content in both the local language and English. This might be a hindrance for some viewers, potentially preventing them from fully benefiting from the videos.

We found that the number of videos available for the pretransplantation preparation period was quite limited. This scarcity of videos on the preparation period, which is one of the most important stages for LDKT, represents a significant deficiency in terms of informing these patients through social media. As experience with KT increases, recipients with more complex conditions are being accepted into transplantation programs. In this regard, especially for patients with urological anomalies, there is a greater involvement of urologists [18]. It is important for urologists to prepare informative videos for patients regarding the pretransplantation preparation and evaluation periods. In terms of video content, the highest number of videos were on the patient experience, and this category had the lowest scores in terms of quality. Thus, the most accessible video category on YouTube may not be effective in providing patients with quality information. Another noteworthy situation is the increase in the availability of videos related to robot-assisted KT (RAKT) on platforms in recent times. This trend is attributed to the advantages provided by RAKT, leading to an increase in its use for KT procedures [19].

We found that some of the most widely viewed videos involved renowned individuals recounting their personal experiences with KT, as well as television shows featuring episodes dedicated to KT. This trend underscores the substantial effort needed to disseminate precise and reliable information to prominent figures in society and popular TV programs. When celebrities who have undergone KT openly share their journey, this not only serves as a source of inspiration but can also impart valuable insights for individuals in the community grappling with end-stage renal failure and contemplating the transplantation route. However, a notable point is that videos uploaded by patients or TV shows/programs received lower scores than videos uploaded by medical centers or physicians/nurses. This situation underscores the importance of TV shows/programs or celebrities seeking assistance from health care professionals or medical associations for dissemination of accurate information.

Individuals are increasingly using social media and online platforms. The information they obtain in this way influences their opinions and perspectives. Therefore, it is crucial that accurate information is uploaded. Our study revealed that the quality of YouTube videos on LDKT was inadequate. To prevent the negative effects of insufficient and misleading data in clinical practice, it is essential that well-prepared videos are available online, particularly on the widely used platforms such as YouTube.

Our study is not without limitations. One crucial issue is that we evaluated only YouTube videos from a specific date, focusing solely on predetermined keywords. It is important to acknowledge that patients have the freedom to explore various platforms and use different keywords beyond the scope of our study, which could potentially yield different results. Despite this limitation, the popularity of YouTube and the inclusion of two distinct keywords in our study design underscore its significance. Another limitation is that we only considered videos with English audio for the study. Considering the abundance of videos in Hindi or Urdu in particular, individuals proficient in these languages could benefit from other videos as well, and it should be noted that these are not included in our study. Third, we only reviewed videos with more than 10 000 views. While this could be considered a limitation of our study, we believe that the number of views is a criterion that individuals apply when deciding on their video preferences, and we wanted to evaluate the most preferred videos according to the data available. Similarly, the fact that only videos of 4–20 min in length were evaluated can be considered a limitation. However, previous studies applied similar criteria for both the view count and video length [20], [21], [22]. Finally, we evaluated videos that were available at the time. There is a possibility that the creators may remove these videos from YouTube, in which case the quality of the videos available on the platform could change. Beyond these limitations, our research represents a pioneering effort in evaluating web-based educational videos on LDKT. This distinction in itself is a notable contribution to the literature. However, our findings highlight the need to ensure that this patient population has access to a broader array of detailed and accurate videos.

## 5 Conclusions

We found that YouTube videos on LDKT are inadequate for patients seeking accurate information. Numerous videos feature substandard content and disseminate misinformation. Given the increasing prevalence of social media use, health care providers and organizations should develop patient education materials that offer accurate information about LDKT. These materials should be uploaded to popular social media platforms to ensure widespread accessibility and dissemination of correct information to patients.

  ***Author contributions:*** Hakan Bahadir Haberal had full access to all the data in the study and takes responsibility for the integrity of the data and the accuracy of the data analysis.

  *Study concept and design*: Haberal, Piana, Donmez, Territo.

*Acquisition of data*: Haberal, Piana.

*Analysis and interpretation of data*: Haberal, Piana, Bañuelos Marco, Casadevall Rubau.

*Drafting of the manuscript*: Haberal, Piana, Donmez.

*Critical revision of the manuscript for important intellectual content*: Pecoraro, Bañuelos Marco, Prudhomme, López-Abad, Breda, Territo.

*Statistical analysis*: Haberal, Piana, López-Abad, Casadevall Rubau.

*Obtaining funding*: None.

*Administrative, technical, or material support*: Pecoraro, Prudhomme, López-Abad.

*Supervision*: Breda.

*Other*: None.

  ***Financial disclosures:*** Hakan Bahadir Haberal certifies that all conflicts of interest, including specific financial interests and relationships and affiliations relevant to the subject matter or materials discussed in the manuscript (eg, employment/affiliation, grants or funding, consultancies, honoraria, stock ownership or options, expert testimony, royalties, or patents filed, received, or pending), are the following: None.

  ***Funding/Support and role of the sponsor:*** None.

  ***Data sharing statement:*** The data sets generated and/or analyzed for this study are available from the corresponding author on reasonable request.
